# An improved mosquito electrocuting trap that safely reproduces epidemiologically relevant metrics of mosquito human-feeding behaviours as determined by human landing catch

**DOI:** 10.1186/s12936-016-1513-1

**Published:** 2016-09-13

**Authors:** Nicodem J. Govella, Deodatus F. Maliti, Amos T. Mlwale, John P. Masallu, Nosrat Mirzai, Paul C. D. Johnson, Heather M. Ferguson, Gerry F. Killeen

**Affiliations:** 1 Environmental Health and Ecological Sciences Thematic Group, Coordination Office, Ifakara Health Institute, PO Box 78373, Kiko Avenue, Mikocheni, Dar es Salaam, United Republic of Tanzania; 2 College of Medical, Veterinary and Life Sciences, Boyd Orr Centre for Population and Ecosystem Health, University of Glasgow, Glasgow, UK; 3 Bioelectronics Unit, University of Glasgow, Graham Kerr Building, Glasgow, G12 8QQ UK; 4 Vector Biology Department, Liverpool School of Tropical Medicine, Pembroke Place, Liverpool, L3 5QA UK

**Keywords:** Malaria vector, *Anopheles gambiae*, *Culex*, Mosquito, Behaviour, Exposure-free, TRAPS, Africa

## Abstract

**Background:**

Reliable quantification of mosquito host—seeking behaviours is required to determine the efficacy of vector control methods. For malaria, the gold standard approach remains the risky human landing catch (HLC). Here compare the performance of an improved prototype of the mosquito electrocuting grid trap (MET) as a safer alternative with HLC for measuring malaria vector behaviour in Dar es Salaam, Tanzania.

**Methods:**

Mosquito trapping was conducted at three sites within Dar es Salaam representing a range of urbanicity over a 7-month period (December 2012–July 2013, 168 sampling nights). At each site, sampling was conducted in a block of four houses, with two houses being allocated to HLC and the other to MET on each night of study. Sampling was conducted both indoors and outdoors (from 19:00 to 06:00 each night) at all houses, with trapping method (HLC and MET) being exchanged between pairs of houses at each site using a crossover design.

**Results:**

The MET caught significantly more *Anopheles gambiae* sensu lato than the HLC, both indoors (RR [95 % confidence interval (CI)]) = 1.47 [1.23–1.76], P < 0.0001 and outdoors = 1.38 [1.14–1.67], P < 0.0001). The sensitivity of MET compared with HLC did not detectably change over the course of night for either *An. gambiae* s.l. (OR [CI]) = 1.01 [0.94–1.02], P = 0.27) or *Culex* spp. (OR [CI]) = 0.99 [0.99–1.0], P = 0.17) indoors and declined only slightly outdoors: *An. gambiae* s.l. (OR [CI]) = 0.92 [0.86–0.99], P = 0.04), and *Culex* spp. (OR [CI]) = 0.99 [0.98–0.99], P = 0.03). MET-based estimates of the proportions of mosquitoes caught indoors (*P*_*i*_) or during sleeping hours (*P*_*fl*_), as well as the proportion of human exposure to bites that would otherwise occurs indoors (π_*i*_), were statistically indistinguishable from those based on HLC for *An. gambiae* s.l. (P = 0.43, 0.07 and 0.48, respectively) and *Culex* spp. (P = 0.76, 0.24 and 0.55, respectively).

**Conclusions:**

This improved MET prototype is highly sensitive tool that accurately quantifies epidemiologically-relevant metrics of mosquito biting densities, behaviours and human exposure distribution.

**Electronic supplementary material:**

The online version of this article (doi:10.1186/s12936-016-1513-1) contains supplementary material, which is available to authorized users.

## Background

Mosquito-biting behaviour plays an essential role in determining not only where and when vector-borne disease transmission occurs, but also in assessing the level of impact that can be reasonably expected of specific vector control interventions [1, 2]. Malaria vector species exhibit diverse feeding behaviours: some feed predominantly indoors and late at night while others bite mostly outdoors in the evening and early morning [3–12]. While behaviour characterization of *Culex* spp, especially the abundant populations of *Culex quinquefasciatus* that proliferate and transmit lymphatic filariasis in urban settings [13, 14], is rarely documented, this mosquito species may also exhibit diverse biting behaviour [15].

Measuring the timing and location of human exposure to mosquito bites is therefore essential for designing and selecting appropriate vector control strategies [2, 9, 16–19]. For example, the use of indoor-based control methods, such as long-lasting insecticidal nets (LLINs) and indoor residual spraying (IRS) are likely to have maximum effect against vectors that feed and rest indoors, such as *Anopheles gambiae* [17, 18, 20, 21], but will be less likely to reduce transmission by vectors such as *Anopheles balabacensis* that primarily feed outdoors in the evening hours before people go to bed [22]. Biting indoors, late at night when people are asleep is therefore the mosquito behaviour that is targeted by the use of LLINs [23], while IRS targets mosquitoes when they rest indoors [24]. Indeed this is why these interventions have drastically impacted malaria transmission across sub-Saharan Africa where the most important vectors exhibit both of these behaviours [25, 26]. These interventions have also contributed to the massive reduction of lymphatic filariasis [27, 28]. The wide-scale use of interventions that selectively target vectors with specific feeding behaviours (e.g., indoor, late night biting with LLINs) is thought to be responsible for shifts in species composition and distribution of biting behaviours. For example, shifts from endophagic (indoor biting) to exophagic (outdoor biting), late to evening biting and changes in species composition that have been observed in some African settings [29–33] and beyond [8, 34, 35]. Indeed the persecution pressure exerted by LLIN and IRS have also been hypothesized to drive selection within individual vector species for heritably altered behaviours [33], such as the changes observed within *Anopheles funestus* [4, 9], which are difficult to explain on the basis of phenotypic plasticity alone [36].

This potential for vector control methods to drive ecological and evolutionary changes in mosquito vector behaviour could undermine strategies that are currently very effective [18, 33, 35]. Thus, there is an urgent need to develop robust sampling tools that can monitor long-term trends in mosquito behaviour and how they respond to interventions.

Currently there are several sampling tools available for monitoring the host-seeking biting densities and associated infection rates of malaria vectors, which include the Centers for Disease Control and Prevention miniature light trap (CDC-LT) [37, 38], Ifakara Tent Trap [39–42] and Mbita trap [43, 44]. While these parameters play an essential role in understanding variations in human exposure hazard, reliable and consistent measurement of other key epidemiologically relevant, malaria vector behaviours (e.g., distribution of bites across different times of the night, or indoor versus outdoor locations) [9, 17, 18, 21, 45, 46] remains only possible with the human landing catch (HLC) gold standard method [38, 47, 48]. For example, even CDC-LT which are widely used for monitoring malaria vector mosquito biting densities, species composition and transmission intensity inside houses vectors across malaria endemic settings [37, 47, 49, 50], studies of the efficacy of CDC-LT for catching malaria vectors outdoors is limited to only few places in Africa [51–53], and our experience of east African settings indicates they catch very few mosquitoes when placed outdoors.

Although the HLC is widely viewed as providing the best representation of human exposure to mosquito bites [9, 17, 18, 21, 45, 46], this method is not without limitations. The number of mosquitoes caught with this method can vary significantly between collectors, likely as a result of variation in their skill and degree of alertness [37, 54–56]. An additional concern is the ethical dilemma arising from the requirement of the HLC to expose collectors to potentially infected mosquito bites [47, 48, 57]. Whilst these risks can be minimized by providing collectors with anti-malarial chemoprophylaxis, in which case participants may be safer from malaria than they would normally be [58], concerns with respect to other vector-borne pathogens such as lymphatic filariasis, dengue fever, and other arboviruses remain [14, 59–62].

Alternative methods which do not require human exposure to mosquito bites, and are sufficiently sensitive and accurate to measure key mosquito-biting behaviour metrics, which determine the choice and impact of vector controls are currently lacking but urgently needed. For example, the proportion of human exposure occurring indoors (π_i_) is an invaluable indicator of how much exposure an LLIN or mosquito-proofed housing may be realistically expected to prevent, as well the extent to which these measures may suppress mosquito human feeding frequency, survival, density and transmission capacity at population level [2, 9, 17–19, 63–65]. Indeed personal estimates of this behavioural metric for individual humans, based on questionnaire surveys of when they went indoors for the evening and left the house in the morning combined with local HLC surveys of mosquito activity, have recently been confirmed as strong epidemiological predictors of malaria infection risk in an urban African settings [65]. It is also noteworthy that it was Garret-Jones himself, who first coined the term *epidemiological entomology* [66], who first began adjusting biting exposure estimates to allow for changing distributions of humans across indoor and outdoor environments in the same way that the proportion of human exposure occurring indoors is calculated today [21].

A series of sequential prototype mosquito electrocuting grid traps, specifically designed to measure these specific metrics of mosquito human-feeding behaviour have therefore been developed and evaluated in previous proof-of-principle studies in Tanzania [67, 68]. In principle, these operate in a similar fashion to HLC by placing electrocuting grids around a human bait host to kill mosquitoes attempting to attack, whereas in HLC they are manually aspirated when they actually land on exposed limbs of the volunteer. While the first evaluation using a commercially available insect-zapping device [67] demonstrated malaria vectors could be captured with reasonable sensitivity, mosquito specimens obtained were often damaged and difficult to identify morphologically [67]. Also the sensitivity of this earlier version, relative to HLC, dropped over the course of the night for a variety of possible technical reasons, limiting their accuracy for measuring patterns of mosquito activity and human exposure because both were consequently skewed to exaggerate biting rates in the early evening [67]. Subsequent studies [68] expanded on these early experiences by developing a custom-engineered mosquito electrocuting trap (MET) that uses a novel, electrical output system, specifically designed to kill mosquitoes without burning the specimens, so that they remain intact for morphological and molecular identification. This MET prototype proved to have encouraging levels of sensitivity relative to HLC, especially when the trap was placed outdoors, and all specimens proved suitable for morphological identification and molecular analysis [68]. However, a number of technical problems with electrical delivery system and durability were reported for this prototype, which limited its ability to consistently reproduce HLC-derived estimates for the proportion of mosquito caught when most humans are indoors (*P*_*fl*_*)* and the proportion of human biting exposure occurring indoors, (π_*i*_) [68].

Based on difficulties reported for this initial MET prototype, this article report the first full field evaluation of the performance of an improved MET prototype, which was redesigned based on lessons learned from these earlier iterations [68]. This improved MET prototype was evaluated, in terms of: (1) its ability to consistently reproduce HLC-derived estimates for key metrics of human-biting behaviours of malaria vectors, and (2) improved catch sensitivity relative to HLC.

## Methods

### Study area and experimental sites

The study was conducted in Dar es Salaam, the biggest city and commercial hub in Tanzania with population of 4.36 million people [69]. Historically, malaria transmission in Dar es Salaam has been stable but at a low level, with an entomological inoculation rates of just over one infectious bites per person per year [5, 65, 70]. However, by the time of this study, at the end of 2012 and the beginning of 2013, malaria transmission rates in Dar es Salaam had been reduced to entomological inoculation rates of fewer than 0.1 infectious bites per person per year as a results of high coverage with LLINs [71], house window screening, sealed eaves or ceilings [72], and regular application of biological larvicides [65]. Spending even 1 or 2 extra hours outdoors in the evening was predictive of malaria risk in Dar es Salaam, presumably due to an appreciable degree of outdoor and early evening biting [5, 63] exhibited by vector populations in the city than is typical for African vectors [17, 18]. Detailed descriptions of the area are available elsewhere [65, 73, 74]. Malaria vectors from the *An. gambiae* sensu *latu* species complex (consisting of *An. gambiae* s.s, *Anopheles arabiensis, Anopheles merus*) and *An. funestus* are present in Dar es Salaam with *An. gambiae* s.s. being responsible for the majority of transmission [5]. *Anopheles arabiensis* in particular tend to bite outdoor at dusk, so at least half of the biting exposure to this species occurs outdoors [63]. Although the peak-biting times of *An. gambiae* s.s. remain approximately consistent with those of classical reports [75], it does prefer to feed outdoors [5, 63]. *Culex* spp., especially *Culex quinquefasciatus,* are far more abundant, accounting for more than 95 % of all mosquitoes in Dar es Salaam [5, 42, 76, 77]. In addition to transmitting lymphatic filariasis and a variety of other pathogens, these species cause significant nuisance in Dar es Salaam and elsewhere [13, 78, 79]. Previous surveys of *Culex* spp. behaviours in Dar es Salaam reveal a strong preference for feeding outdoors, with an activity period that spans the entire night, much of which occurring when people sleep, so that slightly more than half of human exposure occurs indoors in the absence of protective bed nets or mosquito-proofed housing [67].

**Fig. 1:**
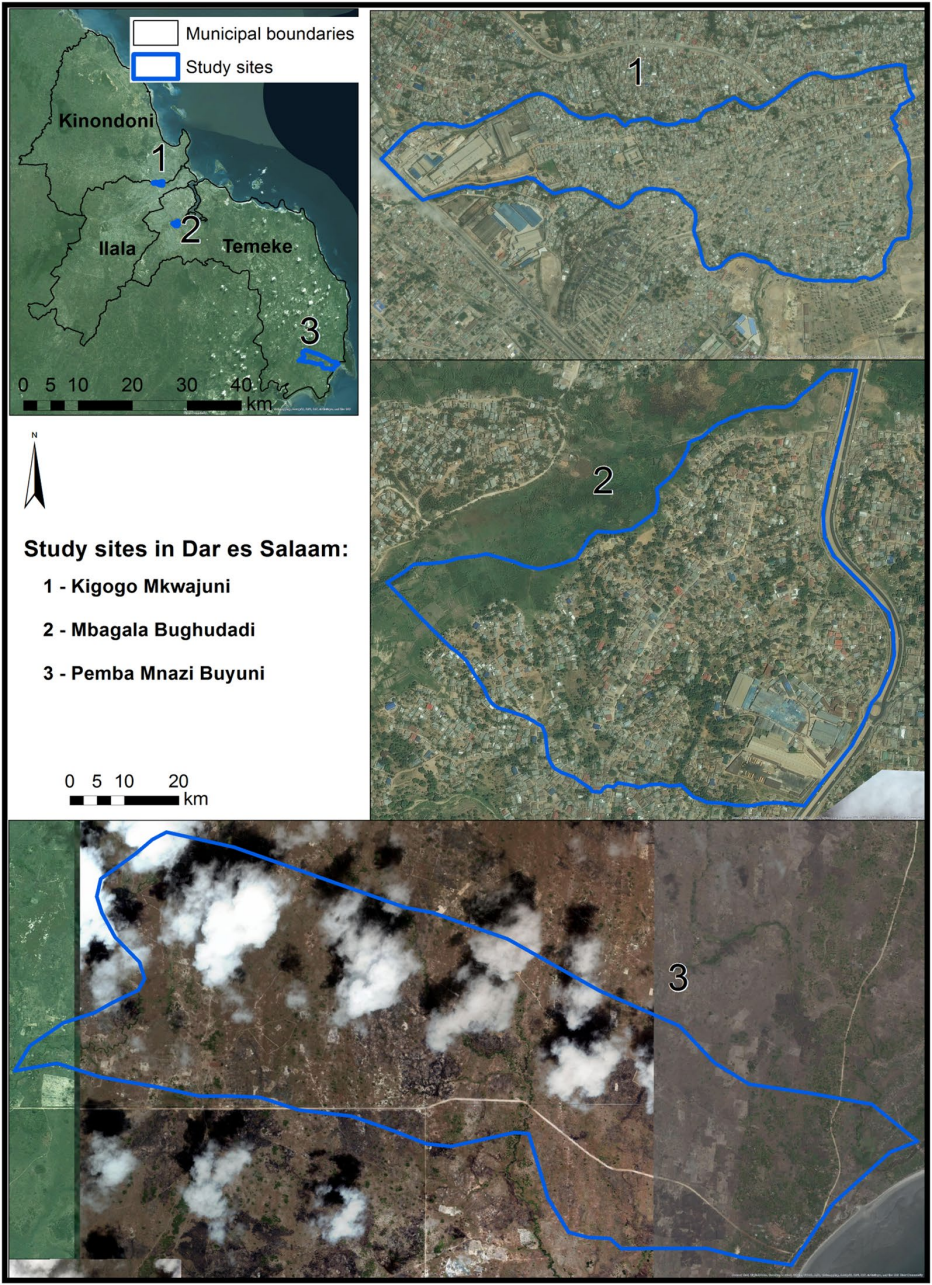
Study area and administrative units in Dar es Salaam. Administratively, Dar es Salaam consists of three municipalities: Kinondoni, Ilala and Temeke. The map highlights three study sites (Kigogo Mkwajuni (urban), Mbagala Bughudag (peri-urban) and Pemba Mnazi Buyuni (rural) where sampling of mosquitoes were carried out

Within Dar es Salaam, three areas representing different levels of urbanization (Kigogo Mkwajuni (urban), Mbagala Bughudad (semi-urban) and Pemba Mnazi Buyuni (rural) (Fig. 1) with detectable levels of *An. gambiae* s.l. were selected as study location. Factors used in the classification of these selected sites included: geographical overview of the area, population density, land use type, socio-economic status, and based on people’s experience. Site selection was also guided by the necessity for sufficient malaria vector mosquito densities, so that it was possible to catch sufficient numbers to measure their biting behaviour. While Kigogo Mkwajuni (urban) and Mbagala Bughudadi (semi-urban) are both densely populated, these are informal, unplanned settlements, bordering rivers that regularly flood during the rainy season. While Mbagala Bughudadi is at the southern edge of the city along the Mbagala river, Kigogo Mkwajuni is located very centrally at the edge of the Msimbazi river valley, the largest flood plain in the city. Pemba Mnazi, although administratively part of the Dar es Salaam city region, is very rural in character, with only a few, small, scattered houses, some of them with thatched roofs (Fig. 1). It is approximately 70 km southeast of Dar es Salaam, where fishing with some agriculture are the main income-generating activities.

### Mosquito trapping methods

#### Mosquito electrocuting grids (MET)

**Fig. 2:**
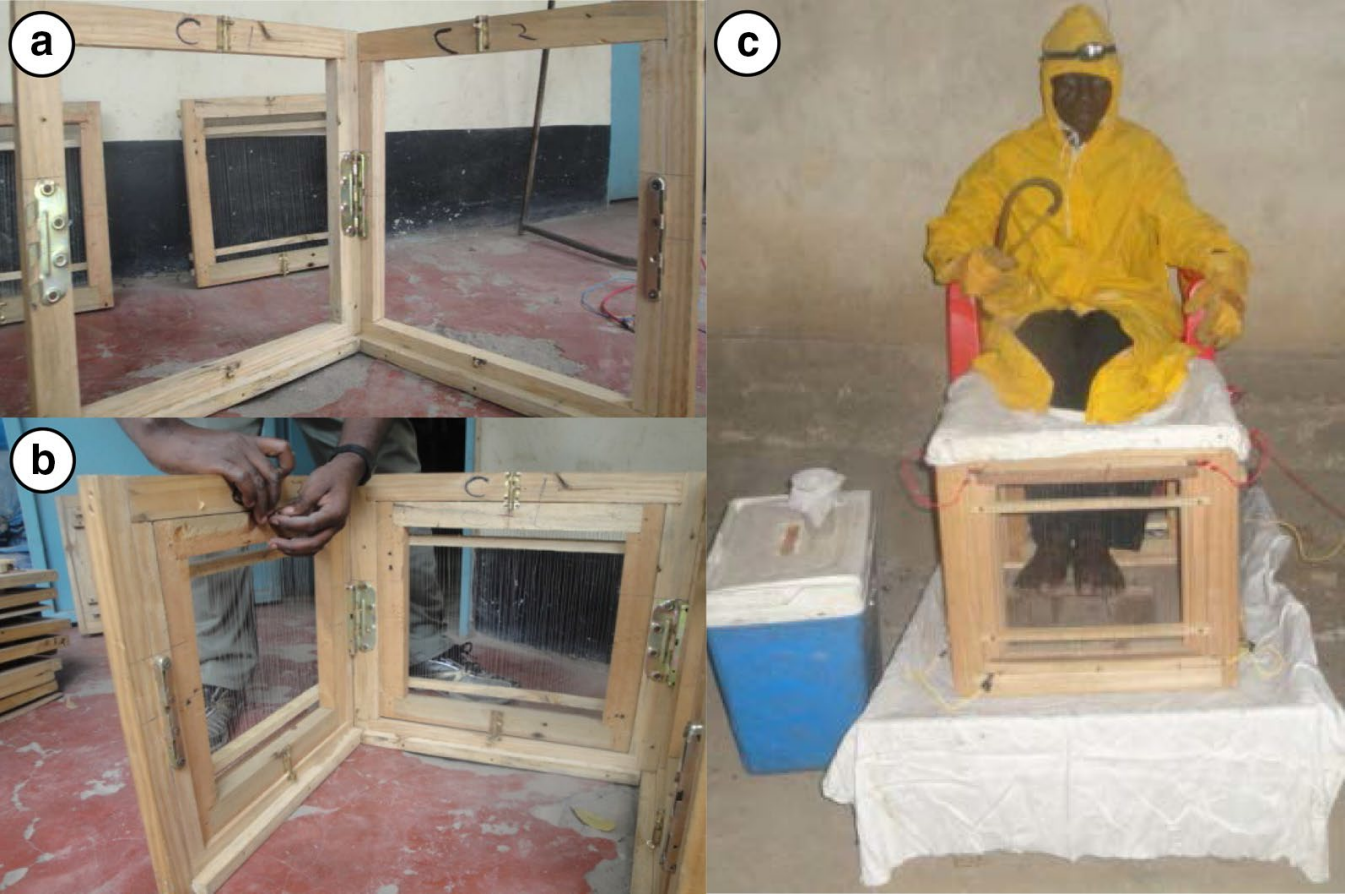
Step-wise setting and improvement made to the mosquito electrocuting trap (MET). **a** Locking together of hinges connecting individual panels with bolts; **b** locking of assembled panels into the main, outer frame; **c** fully assembled MET in use by a human participant wearing protective clothes except for on his feet, which are placed within the MET frame

The MET is composed of four wooden panel frames measuring 35 × 35 cm, arranged to form a square cavity into which human volunteers’ legs are placed (Fig. 2c). The panels hold sets of vertical parallel stainless steel wires spaced 5 mm apart, which are electrically connected to a 24 V battery-powered stable direct current (DC) power source, thereby creating an electric potential between the wires, which is sufficient enough to kill mosquitoes trying to pass through the wires, but without destroying the specimen, as observed with previous prototypes [68]. The power is supplied at low output, which is sufficient to kill mosquitoes on contact but poses no harm if accidentally contacted by volunteer. This combination of voltage with current setting was identified through pilot laboratory experiments using insectary-reared *An. gambiae* and *An. arabiensis* specimens with an a prior minimum kill probability threshold of 80 % [80]. The MET prototype used was modified to improve upon shortcomings reported in an earlier version which included the tendency to short circuit and weak physical stability [68]. Specific changes were: (1) introduction of hinges to secure the four angles of the main frame (Fig. 2a), and (2) better alignment of grid wires into the frame using grooves which minimized the possibility of opposing wires contacting each other and short circuiting. During mosquito trapping, each MET unit was placed on a 2 m × 2 m wooden frame platform placed on a white sheet (Fig. 2c) which made it easier for collectors to see the electrocuted mosquitoes that dropped on the floor. The four legs of the platform were placed in water bowls to create a barrier that prevented ants from crawling onto it and consuming dead mosquito samples. During mosquito collection, a volunteer sits with their lower limbs placed inside the square trapping box (Fig. 2c) to act as attractive bait. Mosquitoes were captured by a single adult male per location using a MET over a 12-h period on each night of experiments (18:00–06:00 h). Sampling was conducted for 45 min of each hour, followed by a 15-min break period during which the trap was turned off, and mosquitoes collected either from the floor of the platform where they had fallen after electrocution, or from the grid panel surfaces using forceps. This 15-min break also allowed for exchange of collectors between matched indoor and outdoor stations at each house after each hour.

### Human landing catch (HLC)

To do a HLC, a single male adult volunteer exposed his legs and collected mosquitoes upon landing on his legs with a mouth aspirator as previously described [38, 54, 55, 81]. Similar to MET, sampling here was also conducted at each sampling location for 45 min of each hour, from 18:00 to 06:00, allowing 15-min breaks for rest and refreshment, and for exchange of collectors between matched indoor and outdoor location at each house.

### Experimental design

Within each of the three study sites described above, and in Fig. 1, a block (site) of four houses with open eaves, all of which were all at least 50 m from each other, were purposively selected for entomological survey by HLC and MET. At each house, a corresponding outdoor-catching station was established approximately 5 m outside the assigned house with a raised platform and plastic sheeting roof to protect against rain, exactly as previously described [67]. In all houses, the indoor mosquito-capture stations were set up within the living room. On each night of experiments, mosquitoes were sampled both indoors and outdoors at all four houses at a given site. On the first night of sampling, two of the four houses were randomly allocated for sampling using HLC, and the remaining two allocated to MET collection. On the second night of experimentation, the two capture methods were exchanged between houses (e.g., MET sampling was conducted at houses where HLC had been done previously and vice versa) such that all methods were used at all four houses in a two-day period (thus completing one replicate) of crossover design. A specific pair of volunteers was assigned to each household (one for indoor and outdoor sampling, respectively) and remained there for the two-night replicate to ensure that only sampling techniques and not volunteers were exchanged between houses. However, each pair of volunteers were swapped between the indoor and outdoor catching stations after each hour to minimize systematic bias due to differential attractiveness and collection skill of collectors. After each two-night survey replicate at a single site, the experiment moved to the next site for implementation of another replicate with the same crossover design. These two-night survey replicates were rotated through all three sites over a total of six nights of sampling within a single working week to complete one full round of experimental replication. This weekly replication cycle of experimentation was conducted from 17 December, 2012 to 4 July, 2013, over a total of 168 nights of sampling and 28 replication weeks.

### Processing of samples

Mosquito samples from all catches were first sorted, counted and morphologically identified as either *An. gambiae* s.l., *An. funestus* [75, 82] or *Culex* spp. with the aid of a stereomicroscope. All *An. gambiae* s.l. were stored in 1.5-mL tubes containing desiccated silica gel under cotton wool for subsequent polymerase chain reaction (PCR) assay [83] to determine sibling species within the complex and enzyme-linked immunosorbent assay (ELISA) [84, 85] for sporozoite infection identification.

### Data analysis

Only *An. gambiae* s.l. and *Culex* spp. were collected in appreciable numbers by this study. Although *An. funestus* is an important malaria vector in Africa, it is now rare in Dar es Salaam and only three specimens were collected in this study, so no detailed analysis of this species was possible. Given the low numbers of *An. gambiae* s.l. specimens caught, and the high proportion of specimens whose DNA failed to amplify in PCR analysis, separate statistical analyses for each sibling species was not possible. While the problem of low DNA amplification rates was consistent across trapping methods by Chi square test, the underlying reasons for poor amplification is suspected to be linked with elevated air temperature in the laboratory. The laboratory air conditioner was out of order at the time when this PCR analyses was conducted. Analysis was, therefore, conducted on *An. gambiae* s.l. as a single taxon, based on counts and derived proportions of mosquitoes identified to complex level using morphological criteria. The other major mosquitoes taxon of interest, as vectors of lymphatic filariasis and other pathogens and as the major cause of biting nuisance in Dar es Salaam and many other African urban centres, were *Culex* spp, which were identified to genus only and correspondingly analysed as a single taxon. Generalized linear mixed effects model (GLMM), allowing for important sources of variance that are not of direct interest were used for all analyses, using R open source statistical software (version Rx 64 2.15.2) augmented with the *lme4* package.

### Catching sensitivity in alternative trap relative to human landing catch

To evaluate the relative sensitivity of the MET, the total of either *An. gambiae* s.l. or *Culex* spp. catch per night was treated as the dependent variable, with trapping method (MET versus HLC) treated as a fixed, categorical variable, with house, participant nested within site, and night of sampling fit as random effects. Since the observations were count data and were not normally distributed, models were fitted using a Poisson distribution. The effect estimates were obtained by exponential transformations of the parameter estimates obtained with this logarithmic link function. Initially, indoor and outdoor catches were analysed separately. Thereafter a similar model was constructed combining both indoor and outdoor data. Additionally, a model that included an interaction term to test for and quantify the effect of any interaction between trap and location (indoor versus outdoor) was fitted so as to check whether the sampling sensitivity of MET relative to HLC is influenced by trap location.

### Effect of hour of night (time) on sampling efficiency of MET

To test whether the sensitivity of MET declined with time over the course of 12 h of collection each night, data were first aggregated to obtain total catches of each mosquito taxon from MET, and from MET plus HLC combined for each site and house on each night, separately calculated for each hour (*h*) in the nightly survey sequence (*h* = 1 for 18:00–19:00, *h* = 2 for 19:00–20:00, *h* = 3 for 20:00–21:00, *h* = 4 for 21:00–22:00, *h* = 5 for 22:00–23:00, *h* = 6 for 23:00–24:00, *h* = 7 for 24:00–01:00, *h* = 8 for 01:00–02:00, *h* = 9 for 02:00–03:00, *h* = 10 for 03:00–04:00, *h* = 11 for 04:00–05:00 and *h* = 12 for 05:00–06:00). Indoor and outdoor collections were analysed separately. The proportion of mosquitoes that were captured with the MET (P_MET_ = MET/(MET + HLC) was treated as the dependent variable with a binomial distribution and logit link function in a GLMM with the sequence hour (*h*) included as a continuous independent variable, and house nested within site as well as sampling night treated as random effects.

### Density dependence

Two mosquito traps are said to exhibit density-dependence (DD) if their relative sampling sensitivity varies with mosquito density, and density-independence (DI) if their relative sampling sensitivity is constant. Graphically, DI can be represented as a linear correlation between the two traps in catches taken across differing densities, and DD as a deviation from linear correlation. Mathematically, two traps show DI if E(*x*_*i*_) = *α*E(*y*_*i*_), where *x*_*i*_ and *y*_*i*_ are the *i*th of *n* paired mosquito catches from traps X and Y, respectively, E(*x*_*i*_) and E(*y*_*i*_) are the expected counts of *x*_*i*_ and *y*_*i*_, and *α* is a scaling constant. DD can be modelled as following a power law, E(*x*_*i*_) = *α*E(*y*_*i*_)^*β*^, where the exponent *β* governs the degree of non-linearity and therefore the degree of DD [53, 86]. DI is therefore a special case of DD where *β* = 1, so the extend of DD can be assessed as deviation of an estimate of *β* from 1. Estimation of *β* by regression of *y* on *x*, or vice versa, would give biased results when neither trap is an error-free measure of mosquito density. Instead we modelled the *n* paired catches as reflecting variation in underlying mosquito density, *z*_*i*_, which was taken to be a log-normally distributed latent variable, with $\log \left( z \right)\sim N(0, \sigma_{z}^{2} )$. The expected values of *x*_*i*_ and *y*_*i*_ were modelled as ${\text{E}}\left( {x_{i} } \right) = \alpha_{x} z_{i}^{\surd \beta }$ and ${\text{E}}\left( {y_{i} } \right) = \alpha_{y} z_{i}^{ 1/\surd \beta }$. By solving both equations for *z*_*i*_ it can be shown that ${\text{E}}(x_{i} ) = \frac{{\alpha_{x} }}{{\alpha_{y}^{\beta } }}{\text{E}}(y_{i} )^{\beta }$ and ${\text{E}}(y_{i} ) = \frac{{\alpha_{y} }}{{\alpha_{x}^{1/\beta } }}{\text{E}}(x_{i} )^{1/\beta }$, so that ${\text{E}}(x_{i} ) = \frac{{\alpha_{x} }}{{\alpha_{y}^{\beta } }}{\text{E}}(y_{i} )^{\beta }$ and ${\text{E}}(y_{i} ) = \frac{{\alpha_{y} }}{{\alpha_{x}^{1/\beta } }}{\text{E}}(x_{i} )^{1/\beta }$, giving a power law relationship between the expected densities of traps X and Y with exponent *β*, so that E(*x*_*i*_) ∝ E(*y*_*i*_)^*β*^ and E(*y*_*i*_) ∝ E(*x*_*i*_)^1/*β*^. The observed counts, *x*_*i*_ and *y*_*i*_, were assumed to be drawn from a negative binomial distribution such that *x*_*i*_ ∼ *NB*(*λ*_*xi*_, *θ*) and *y*_*i*_ ∼ *NB*(*λ*_*yi*_, *θ*), using the parameterisation of the negative binomial with mean *λ* and variance *λ* + *λ*^2^/*θ*. The dispersion parameter *θ* is inversely related to trap reliability, lower values of *θ* corresponding to higher levels of over-dispersion in the X and Y catches, and consequently weaker correlation. This method differs from existing methods [53, 86] by incorporating symmetry between the traps and by modelling overdispersion. It can be shown that the DI model is equivalent to a negative binomial GLMM with an indicator for trap type centred on zero fitted as a fixed effect and log(*z*_*i*_) being a normally distributed random effect. This GLMM can be extended to DD by allowing the X:Y ratio of random effect standard deviations, which is *β*, to differ from 1. The DD GLMM is therefore the DI GLMM extended to include random slopes, with the inter-trap random effects correlation set to 1. It should, therefore, be possible to fit the DI and DD models using standard maximum likelihood methods for GLMMs. However, we used MCMC in the program JAGS [87, 88] because of the ease of obtaining credible intervals (CI) for the model parameters. The extent of deviation from DI was gauged by estimating *β* from the DD model, while the strength of linear correlation between the X and Y catches was calculated from the log-scale variance components estimated from the DI model as $r = {\text{Cov}}(x,y)/\sqrt {{\text{Var}}\left( x \right){\text{Var}}(y)}$, where ${\text{Cov}}\left( {x, y} \right) = \sigma_{z}^{ 2} ,{\text{ Var}}\left( x \right) = {\text{Cov}}\left( {x, y} \right) + \psi^{( 1)} \left( \theta \right) + { \log }\left( { 1 + 1/\alpha_{x} } \right)$, and Var(*y*) was calculated by replacing *α*_*x*_ with *α*_*y*_ in the formula for Var(*x*). ψ^(1)^(*θ*) approximates the variance from the gamma component of the negative binomial distribution, where ψ^(1)^ represents the trigamma function [89], and $\frac{1}{n}\mathop \sum \nolimits { \log }[1 + 1/{\text{E}}\left( {x_{i} } \right)]$ approximates the variance from the Poisson component appendix 1 of [90]. Because ψ^(1)^(*θ*) ≈ 1/*θ*, higher values of *θ*, which correspond to lower levels of overdispersion, will also correspond with higher values of *r*_*xy*_, in line with intuition. This method was used to assess density dependence between MET (taken to be Y) and HLC (taken to be X). Estimates and 95 % CIs for *β* and *r* were calculated as mean and 2.5 and 97.5 % centiles from 5 × 10^5^ MCMC samples from the posterior distribution following 10^5^ burn-in iterations. The effective MCMC sample size for all parameters was >2000. Prior distributions for log(*α*_*x*_), log(*α*_*y*_) and log(*β*) were normal with means of zero and variances of 10^4^, and the prior distributions for log(*θ*) and log(*σ*_*z*_^2^) were uniform from −10 to 10. Note, because multiple traps of the same type were used simultaneously each night, mosquito catches were first aggregated by trap types, night of collection and by hour. This analysis was followed by plotting catches between the two methods.

### Estimating epidemiologically relevant metrics of mosquito behaviours and human exposure patterns

Two key metrics of the behavioural preferences of mosquitoes, as well as another metric of the distribution of human exposure to mosquito bites, were estimated as previously described [17, 21, 67, 68] from the entomological data collected as described above, and combined with questionnaire survey data describing when residents of Dar es Salaam spend their time indoors and outdoors [5]: (1) the proportion of mosquitoes caught indoors (*P*_*i*_), which is obtained by dividing the total number of mosquitoes that were caught indoors by the total caught indoors and outdoors (I_18:00→06:00 h_)/(I_18:00→06:00 h_ + O_18:00→06:00 h_): where I and O represent mosquitoes collected indoors and outdoors, respectively, and subscripts indicate the start and end time of collection period; (2) the proportion of mosquitoes that are caught between the first (*f*) and last (*l*) hours when most (at least 50 %) people were asleep and indoors (*P*_*fl*_), obtained by dividing the total number of mosquitoes caught between 22.00 and 05.00 [5] by the total number of mosquitoes caught over the entire night (I_22:00→05:00 h_ + O_22:00→05:00 h_)/(I_18:00→06:00 h_ + O_18:00→06:00 h_); (3) the proportion of human exposure to mosquito bites that would occur indoors in the absence of personal or household physical protection (π_*i*_), and that can therefore be directly prevented by using a bed net, obtained by dividing the number of mosquitoes that were collected indoors during sleeping hours from 22:00 to 05:00 by itself plus the number collected outdoors outside of sleeping hours from 18:00 to 22:00 plus from 05:00 to 06:00 (I_22:00→05:00 h_)/(I_22:00→05:00 h_ + O_05:00→22:00 h_). Calculation of *P*_*i,*_*P*_*fl*_ and π_*i*_ have been previously described elsewhere [17, 18, 21]. To estimate these metrics of mosquito behaviours, the proportions of mosquitoes caught or the proportions of human exposure to mosquito bites from each taxon were each treated as dependent variables with a binomial distribution and a logit link function in GLMMs [91]. Trap type (MET versus HLC) was fitted as a fixed categorical factor, with participant nested within house and then house nested within site, as well as night of experimentation as random effects, to account for the substantial variance that is typically associated with these nuisance variables in mosquito capture experiment [92–94]. Because multiple traps of the same type were used simultaneously in each experimental night, data were aggregated by sampling night, house, hour, location (indoor *versus* outdoor), site and trap type. These estimate proportions of *P*_*i*_, *P*_*fl,*_ and π_*i*_ were derived from count data as previously described [67].

## Results

A total of 62,202 female mosquitoes were sampled from all three collection sites, of which 96 % (59,814) were *Culex* spp. Of 1373 female anopheline mosquitoes collected, 86 % (1184) were *An. gambiae* s.l., 0.2 % (3) *An. funestus,* and 13.5 % (186) *Anopheles tenebrosus*. Table 1 summarizes the number of mosquitoes from different groups that were collected from each site by the two sampling methods. Because, *An. funestus* and *An. tenebrosus* were collected in very low numbers, they were excluded from further GLMM analysis.

**Table 1:** Number of mosquitoes caught from different sites by two methods and crude estimates of sensitivity of mosquito electrocuting trap (MET) relative to human landing catch (HLC)

Collection sites	Catch per method		Total catch	Relative sensitivity
	MET	HLC		
*Anopheles gambiae* s.l.				
Kigogo Mkwajuni (urban)	102	129	231	0.78
Bughudad (semi-urban)	492	236	728	2.08
Pemba Mnazi (rural)	127	98	225	1.30
Overall catch	721	463	1184	1.56
*Anopheles funestus*				
Kigogo Mkwajuni (urban)	0	0	0	NA
Bughudad (semi-urban)	2	1	3	2
Pemba Mnazi (rural)	0	0	0	NA
Overall catch	2	1	3	NA
*Anopheles tenebrosus*				
Kigogo Mkwajuni (urban)	5	3	8	1.67
Bughudad (semi-urban)	47	64	111	0.73
Pemba Mnazi (rural)	24	43	67	0.58
Overall catch	76	110	186	0.69
*Culex* spp.				
Kigogo Mkwajuni (urban)	10,172	10,986	21,156	0.93
Bughudad (semi-urban)	10,418	11,327	21,745	0.92
Pemba Mnazi (rural)	8338	8573	16,911	0.97
Overall catch	28,928	30,886	59,814	0.94
*Mansonia* sp.				
Kigogo Mkwajuni (urban)	36	28	64	1.29
Bughudad (semi-urban)	315	558	873	0.56
Pemba Mnazi (rural)	32	26	58	1.23
Overall catch	384	612	995	0.63
*Aedes aegypti*				
Kigogo Mkwajuni (urban)	0	0	0	NA
Bughudad (semi-urban)	0	0	0	NA
Pemba Mnazi (rural)	20	0	0	NA
Overall catch	20	0	0	NA

Of the 1172 *An. gambiae* s.l. that were subjected to PCR analysis, only 427 (36 %) were successfully amplified and identified as *An. gambiae* s.s. (136, 31.8 %), *An. arabiensis* (258, 60.4 %) and *An. merus* (33, 8 %). Although amplification success was generally poor, it was consistent for specimens caught with either methods [39 % (236/598) for MET versus 33 % (191/574) for HLC, $\chi^{ 2} = 2. 4 7$, df = 1, P = 0.116]. The MET consistently caught at least a third more *An. gambiae* s.l. than the reference HLC gold standard method but caught slightly less *Culex* spp. (Table 2). No significant difference between indoor versus outdoor locations was detected for the relative capture efficacy of MET compared to HLC, for either *An. gambiae* s.l. (RR = 0.98, P = 0.86) or *Culex* spp. (RR = 0.97, P = 0.15).

**Table 2:** Comparisons of numbers of female *Anopheles gambiae* complex and *Culex* sp. caught between indoors and outdoors by alternative mosquito electrocuting grid (MET) relative to reference human landing catch (HLC), pooling data from each sites and analysed by generalized linear mixed effect model (GLMM)

Collection methods	Trap nights	Total catch	Mean catch	RR [95 % CI]	P
*Anopheles gambiae* s.l.					
*Indoors*					
HLC	308	226	0.73	1^a^	NA
MET	308	348	1.13	1.47 [1.23–1.76]	<0.0001
*Outdoors*					
HLC	308	237	0.77	1^a^	
MET	308	373	1.12	1.38 [1.14–1.67]	<0.0001
*Indoors and outdoors combined*					
HLC	616	463	0.75	1^a^	
MET	616	721	1.17	1.42 [1.24–3.48]	<0.0001
*Culex* spp.					
*Indoors*					
HLC	308	13,613	44.19	1^a^	
MET	308	12,576	40.83	0.93 [0.90–0.95]	<0.0001
*Outdoors*					
HLC	308	17,273	56.08	1^a^	NA
MET	308	16,352	53.09	0.95 [0.93–0.97]	<0.0001
* Indoors and outdoors combined*					
HLC	616	30,886	50.14	1^a^	
MET	616	28,928	46.96	0.94 [0.92–0.95]	<0.0001

*NA* not applicable, *RR* relative rate, *CI* confidence interval

^a^Reference group

**Fig. 3:**
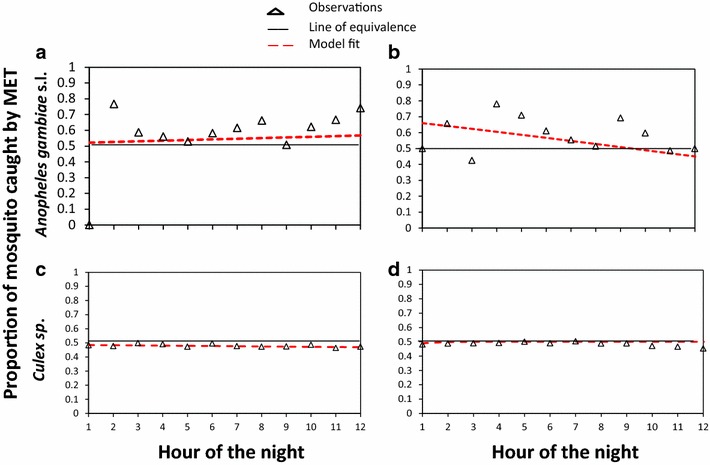
Sensitivity of electrocuting grids trap (MET) relative to human landing catch through the entire time of the night. Relative sensitivity of MET to catch *An. gambiae* s.l. indoor (**a**), *An. gambiae* s.l. outdoor (**b**), *Culex* spp. indoors (**c**) and *Culex* spp. outdoors (**d**) compared to HLC. *Diamond* shows the actual observations, discontinued lines the model fit for the observed values and *solid line* assumes equivalence of sensitivity between MET and HLC overnight

The MET also exhibited strong sampling consistency over the course of the night relative to the HLC (Fig. 3). Its relative sampling efficacy did not detectably change with time over the course of the entire night for both *An. gambiae* s.l. (OR [95 % CI] = 1.01 [0.94–1.02], P = 0.27) and *Culex* spp. (OR [CI] = 0.99 [0.99–1.0], P = 0.17) in indoor environment, but with significant decline in outdoor environment (*An. gambiae* s.l. OR [CI] = 0.92 [0.86–0.99], P = 0.04), and *Culex* spp. (OR [CI] = 0.99 [0.98–0.99], P = 0.03). The size of the effect for such decline was however, not big enough to affect human-vectors interactions behavioural outcome.

**Fig. 4:**
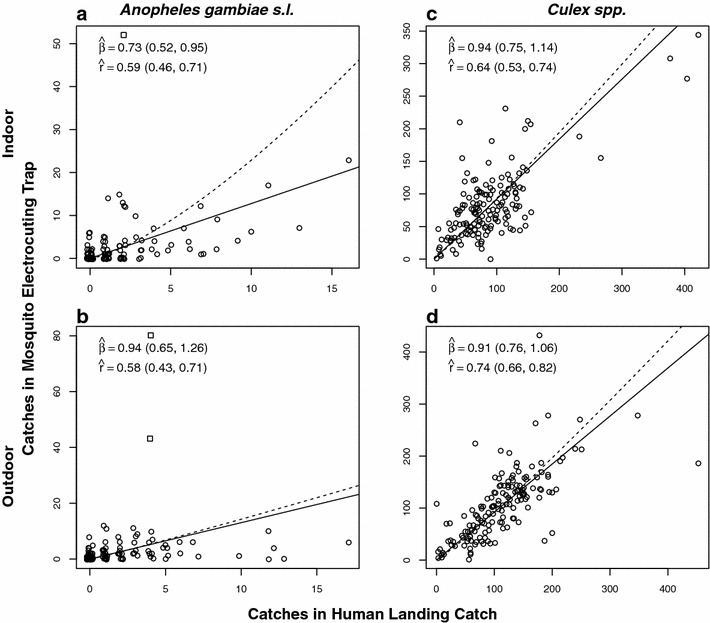
Panels illustrate density-dependence by plotting the mosquito catches in MET against those in HLC. **a**, **b** represent *An. gambiae* s.l. catches indoors and outdoors, respectively, while **c**, **d** represent *Culex* spp. catches indoors and outdoors, respectively. Data points are *open circles*, except for three data points, depicted with *open squares*, which represent high outlier MET catches or low outlier HLC catches (see text for details). Estimates and 95 % CIs are given for the density dependence exponent, ($\hat{\beta }$), and the linear correlation coefficient ($\hat{r}$). Model-predicted relationships are shown between the MET and HLC catches as estimated with either the linear density independence model (*solid line*) and the non-linear density dependence model (*dashed line*)

**Fig. 5:**
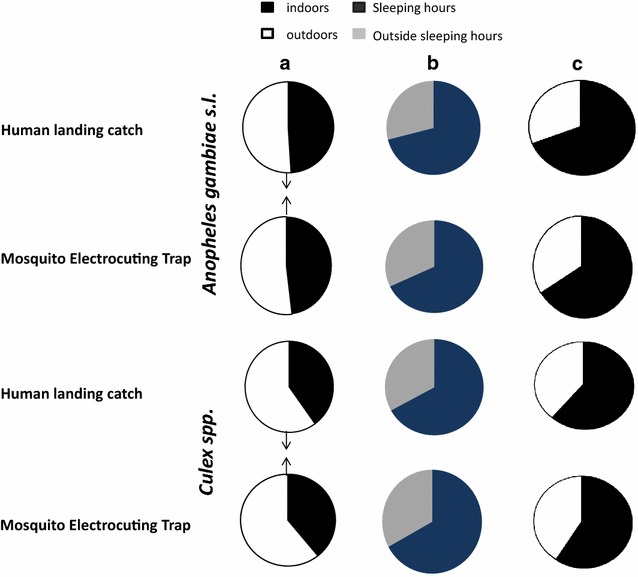
Pie charts illustrating the proportions of mosquitoes caught. Indoors and outdoors (**a**), during sleeping and outside sleeping hours (**b**), human exposure to mosquitoes bites occurring indoors and outdoors (**c**) as estimated by the HLC and MET. The *light areas* represent proportion of mosquitoes caught outdoors and the outdoors human exposure, the *dark areas* represent proportion caught indoors and indoors human exposure, the *dark blue* and *grey areas* represent proportion of mosquitoes caught during sleeping and outside sleeping hours, respectively

Significant density dependence between MET and HLC was detected only for *An. gambiae* s.l. indoors (Fig. 4a). The 95 % CI for the density dependence exponent, *β*, was entirely below one, suggestion that MET sampling sensitivity increases relative to HLC at higher densities ($\hat{\beta }$ [95 % CI] = 0.73 [0.52, 0.95]). There was no evidence of density dependence outdoors ($\hat{\beta }$ [95 % CI] = 0.94 [0.65, 1.26]). However, both data sets contained outlier observations where the MET catch was anomalously high (1 indoors and 2 outdoors with catch >40, Fig. 4a, b). We tested the sensitivity of the *An. gambiae s.l.* results to these outliers by removing them and re-estimating $\beta$ (Additional file 1: Figure S1). The finding of density dependence in *An. gambiae* s.l. indoors proved to be sensitive to the MET outlier ($\hat{\beta }$ [95 % CI] = 0.79 [0.57, 1.04]), while the non-detection of density dependence in the *An. gambiae* s.l. outdoors analysis was unchanged ($\hat{\beta }$ [95 % CI] = 1.17 [0.77, 1.58]). There was no evidence of density dependence in *Culex* spp. indoor ($\hat{\beta }$ [95 % CI] = 0.94 [0.75, 1.14]) or outdoor ($\hat{\beta }$ [95 % CI] = 0.91 [0.76, 1.06]). The wider 95 % CIs in *An. gambiae* s.l. relative to *Culex* spp. suggest that sensitivity to detect density dependence was lower in the former species, probably due to the lower catch numbers. The greater noisiness of the *An. gambiae* s.l. catches was also reflected in lower estimates of linear correlation between the two methods (Fig. 4). *Anopheles gambiae* s.l. gave the lowest correlation estimates ($\hat{r}$ [95 % CI] = 0.59 [0.46, 0.71] indoors, $\hat{r}$ [95 % CI] = 0.58 [0.43, 0.71] outdoors, which were not substantially changed by removing outliers (Additional file 1: Figure S1), while *Culex* spp., where mean catches were considerably higher (Table 2), showed rather higher correlations ($\hat{r}$ [95 % CI] = 0.64 [0.53, 0.74] indoors, [95 % CI] = 0.74 [0.66, 0.82] outdoors). No statistical difference between MET and HLC was apparent in terms of the estimates of proportion of mosquitoes caught indoors (*P*_*i*_), the proportion caught during sleeping hours spent indoors (*P*_*fl*_) or the proportion of human exposure to mosquito bites that occurs indoors (π_*i*_) for either *An. gambiae* s.l. or *Culex* spp. (Table 3). These observed behavioural patterns of mosquito-biting activity linked with human behaviour also appeared descriptively very similar for MET and HLC (Fig. 5). Both *An. gambiae* s.l. and *Culex* spp. show a clear tendency to prefer feeding during sleeping hours of the night (*P*_*fl*_ ≫ 0.5) (Fig. 5b), so that the associated proportions of human exposure occurring indoors were also high (π_*i*_ ≫ 0.5) (Fig. 5c). Although *Culex* spp. showed a preference for feeding after 22:00 when most people were likely to be indoors, they also exhibit exophagic behaviour (*P*_*i*_ = 0.43, and 0.44, respectively, as measured by MET and HLC) (Fig. 5a). Unlike *Culex* spp., *An. gambiae* s.l. can be explained as neither endophagic nor exophagic because it exhibits no strong preference for feeding indoors or outdoors (*P*_*i*_ = 0.48 and 0.49, respectively for MET and HLC) (Fig. 5a).

**Table 3:** Comparison between an alternative mosquito electrocuting grid trap (MET) and human landing catch (HLC) methods in estimating three epidemiologically relevant mosquito behaviours of both female *Anopheles gambiae* complex and *Culex* spp. as analysed using binomial logistic generalized linear mixed effect model (GLMM)

Method	Proportion caught indoors (*Pi*)		Proportion caught during sleeping hours (*Pfl*)		Proportion of human exposure occurring indoors (πi)	
	OR [95 % CI]	P	OR [95 % CI]	P	OR [95 % CI]	P
*Anopheles gambiae*						
HLC	1^a^	NA		NA	1^a^	NA
MET	1.12 [0.84–1.51]	0.43	0.76 [0.56–1.03]	0.07	0.84 [0.51–1.37]	0.48
*Culex* spp.						
HLC	1^a^	NA		NA		NA
MET	0.99 [0.96–1.03]	0.76	1.02 [0.99–1.06]	0.24	1.02 [0.96–1.07]	0.55

^a^Reference group

## Discussion

This improved MET represents the first evaluated sampling device that captures Afrotropical malaria and lymphatic filariasis vectors with efficacy that is comparable to the HLC gold standard method. Similar estimates of mosquito abundance were obtained by the MET and HLC, both indoors and outdoors locations, as well as over the course of the night even in the rain. On account of the MET’s ability to accurately reproduce estimates of mosquito abundance and hourly biting profiles, this trapping method generated estimates of epidemiologically relevant metrics of mosquito behaviour (*P*_*i*_, *P*_*fl*_, π_i_) [17, 21, 67, 68] that were indistinguishable from those obtained by HLC. It is encouraging that this MET performed similarly well for two different mosquito taxa: the *An. gambiae* s.l. and the group of *Culex* spp. that mediate transmission of lymphatic filariasis. These two groups have differing ecological characteristics, with the *An. gambiae* s.l. complex being relatively sparse but efficient vectors of residual malaria transmission [5], while the sundry *Culex* spp. reach very high densities and mediate transmission of lymphatic filariasis in Dar es Salaam [95]. Sampling stability over the course of the night together with consistency sampling efficacy between indoors and outdoors appear to be essential requirements of any trap used to measure distributions of mosquito-biting activity across time of night, so that interactions with human behaviour can be accurately calculated [67]. Even with the prototype described here, there was a decline in sampling efficacy for both mosquito taxa over the course of the night in outdoor environment, these declines were quantitatively modest and appear to have had negligible influence on the estimates for the epidemiologically relevant metrics of interactions between humans and vectors that were measured. The unprecedented stability and consistency of sampling efficacy observed in this study is probably the result of specific modifications made to the trap design, especially the introduction of grooves which prevented the possibility of contact between the two adjacent wires which often caused short circuits in previous prototypes [68]. Also, the introduction of hinges increased the physical stability of trap against buffeting by wind and enabled rapid fixing of the device whenever a defect occurred in one panel during an active sampling experiment. Unlike the previous study [68], here the current flow across traps were closely monitored before the start of sampling and after every 3 h of the night. However, there was one occasion when one MET was found to be operating inadequately noting voltage fluctuation at the voltage amplifier unit, so that the defective panel was identified and replaced immediately with the spare one. While previous evaluations of commercially available electric grids anecdotally attributed declining sensitivity over the course of the night to declining battery charge and the accumulation of burnt mosquito cadavers [67], both of these design concerns were addressed by stabilizing the power supply and modifying the electrical configuration of the MET used here. Another important feature that may have contributed to the MET reproducing HLC-derived estimates for metrics of mosquito behaviour is the fact that the trap operated in a similar way to HLC by capturing mosquitoes exactly when they attack a seated human subject. Like evaluations of previous prototypes of electrocuting traps, the MET here also exhibited some differential capture efficacy with respect to different mosquito taxa. The relative sensitivity of MET was consistently higher for *An. gambiae* s.l. than *Culex* spp.. In the earlier study in rural Kilombero Valley, the relative capture efficacy of that MET was consistently higher for *An. funestus* s.l. than for *An. gambiae* s.l. [68]. Similarly, in a preceding study in urban Dar es Salaam, using commercially available electrocuting grids, the sensitivity was 39, 26 and 32 % for *An. gambiae* s.s., *An. arabiensis* and *Culex* spp., respectively [67]. While such differential sensitivity may be a common property of this sampling device, the relative sensitivity observed in this study was consistently high, being as good as or better than HLC for both *An. gambiae* s.l. and *Culex* spp. regardless of being indoors or outdoors.

Another positive result with respect to MET is the association between numbers of mosquitoes caught by the MET method and the HLC method did not show strong evidence for deviation from linearity. In other words, MET tended to exhibit constant sampling efficiency regardless of density. An exception to this tendency was *An. gambiae* s.l. captured indoors, for which the deviation from linearity was detected, with the sampling efficiency of MET being higher relative to HLC at higher densities. However, the evidence for density dependence in *An. gambiae* s.l. indoors was unreliable, being contingent on a single outlying observation. The lack of strong evidence for DD is also consistent with a previous evaluation of a preceding prototype, in a rural Tanzanian setting where high malaria vector densities provided far greater statistical power [68]. It should also be noted that, the correlation coefficients of these two capturing methods were relatively lower in *An. gambiae* s.l. than in *Culex* spp. (Fig. 4), in line with the noisier *Culex* scatter plots, but nevertheless not close to zero, suggesting that both MET and HLC are sensing substantial variation in underlying *An. gambiae* s.l. density. In addition, given that low-rate count data is intrinsically noisy, it seems likely that a substantial amount of the scatter on the plots is caused by noise intrinsic to both methods when densities are low, rather than simply due to unreliability of MET.

While this prototype version can representatively estimate the three key mosquito host-seeking behavioural metrics mentioned above, this MET design could be enlarged in size to accommodate the whole body of a person or a calf and thus allow measuring of another important epidemiologically relevant indicator of vector-borne disease transmission [96, 97], such as host preference of mosquitoes [98–100]. While this indicator is often measured by examining the blood meal origin of wild mosquitoes, usually collected while resting [98, 99, 101, 102], the derived estimates for the proportion of blood meals obtained from each host type is also depend on the abundance and acceptability of each host species [98, 102–104], rather than just host preference. Estimate of host preference based upon blood meal identification are largely driven by the sampling location and methodology. For example blood meals of mosquitoes collected in houses tend to be biased towards humans [105]. Direct competitive-choice experiments [98] may therefore provide complementary direct estimates of actual host preferences rather than the ultimate outcomes of behavioural processes in the field.

Despite all the advantages listed above, this MET design also has some drawbacks which merit attention during further development: while the mosquito-borne diseases it has been designed for predominantly occur in poorly resourced countries of the tropics, the trap requires batteries that need to be recharged at least every 2 days. However, this limitation could be readily overcome with solar recharging technology, even in isolated African rural settings, similar to recent applications of CDC-light traps [49]. Although the four panels of the trap are interconnected by pre-fixed hinges, so it takes less than 5 min to set-up or disassemble this prototype MET, there is clearly room for further improvements with respect to convenience, integrity and robustness. For example, the design may also benefit from improving the frame materials from wooden to lightweight durable materials, such as polyvinyl chloride, so that it is easy to carry, set up and transport. Note also that while the sample collected from MET are intact and can be identified both morphologically [68] and with molecular genetic methods [67, 68], they may be unsuitable for age determination by dissection [106–108] because they tend to dry up relatively fast. This could perhaps be overcome by using other methods of age determination such as near-infrared spectroscopy, which may work with dry samples [109–111]. Also, the material costs alone for this prototype are of $200 per set, so clearly needs to undergo further modification for mass production and large-scale use.

## Conclusions

This improved MET prototype matches the performance of the gold standard HLC method for measuring mosquito abundance and behaviour in Dar es Salaam. This device appears capable of accurately quantifying not only the level of human transmission exposure occurring indoors and outdoors, but also of other underlying behavioural characteristics of mosquito vector populations that determine the degree to which transmission of malaria [4, 18, 35, 112] and other mosquito-borne diseases, such as lymphatic filariasis [13] are vulnerable to targeting with specific vector control measures. This is the first time that an alternative exposure-free mosquito sampling method to potentially risky HLC has been shown to representatively measure these important metrics of mosquito behaviour and human exposure distribution. So while considerable further optimization and validation across a wider variety of settings and mosquito populations remains to be done, these results are encouraging. Furthermore, this device could have broader applications in a range of insect surveillance and control applications.

## Additional file

10.1186/s12936-016-1513-1 Catches in human landing catch.
