# Supplementary figures and images for: An improved mosquito electrocuting trap that safely reproduces epidemiologically relevant metrics of mosquito human-feeding behaviours as determined by human landing catch
Source: Malar J. 2016 Sep 13;15(1):465. doi: 10.1186/s12936-016-1513-1 (PMC5020444; doi:10.1186/s12936-016-1513-1)

Indoor  
Outdoor

**A** *Anopheles gambiae* s.l.

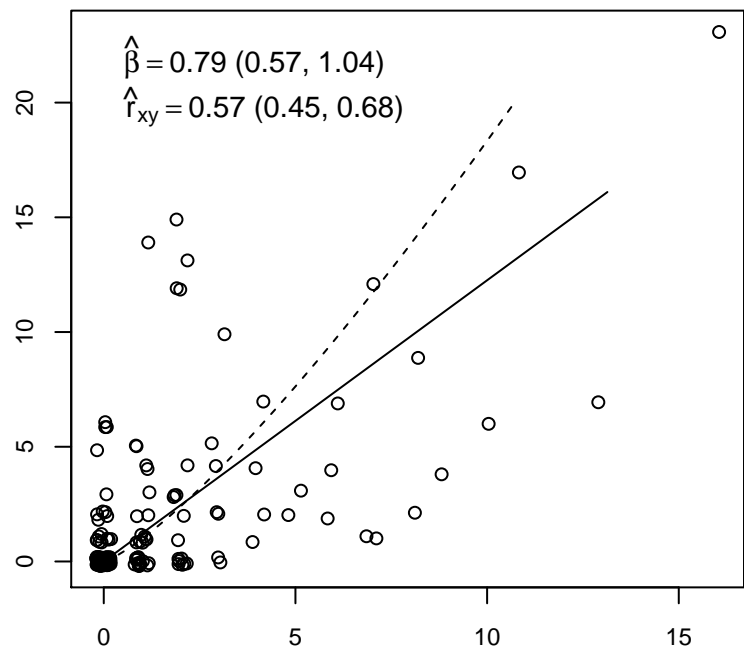

**B**

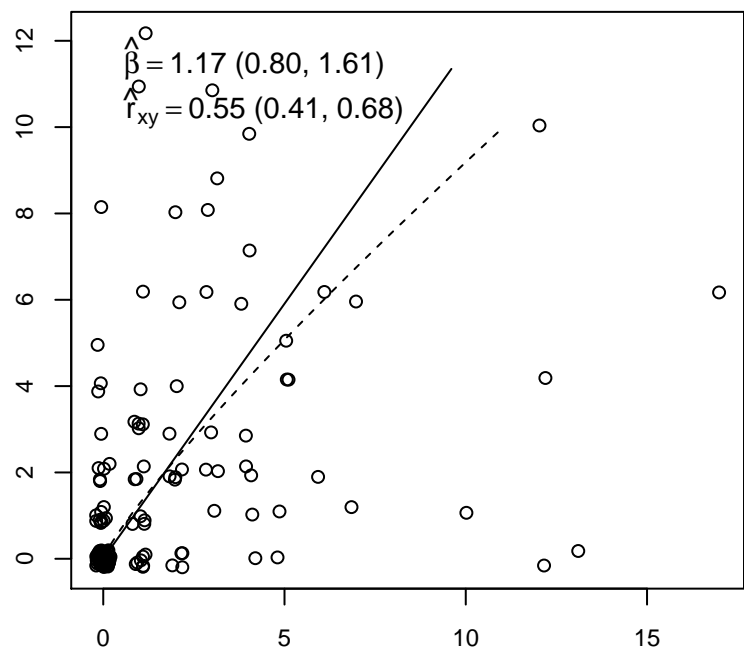

**C** *Culex* spp.

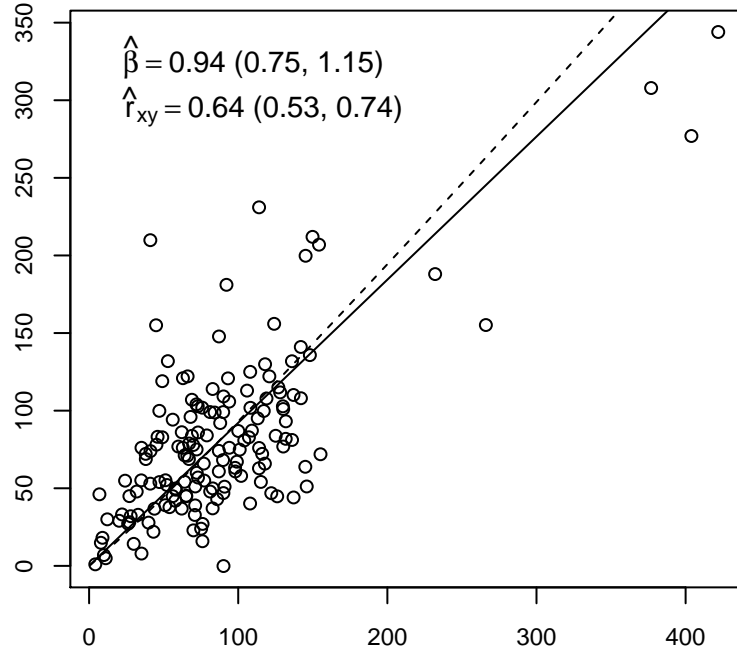

**D**

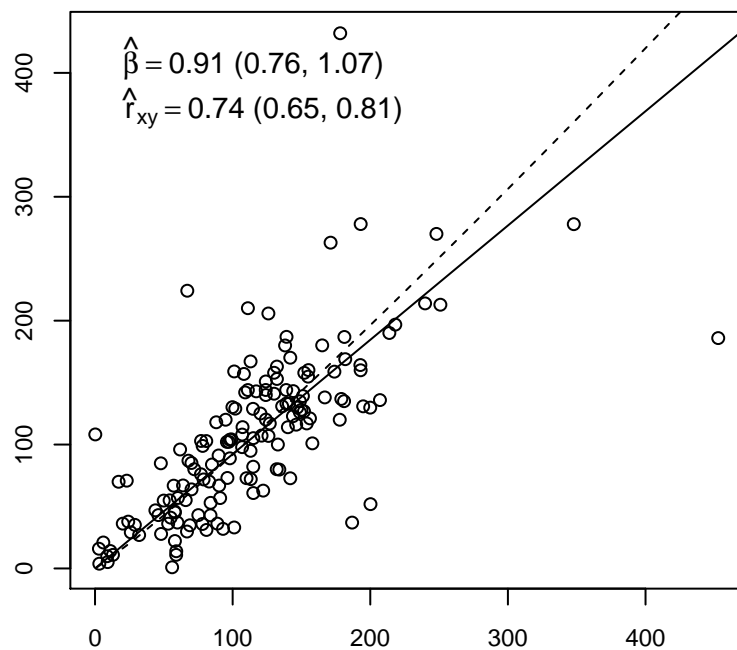

Catches in Human Landing Catch

Supplement: Supplementary file 1 — 10.1186/s12936-016-1513-1 Catches in human landing catch. [file 12936_2016_1513_MOESM1_ESM.pdf]
